# Pre-diagnostic anti-EBV antibodies and primary liver cancer risk: a population-based nested case-control study in southern China

**DOI:** 10.1186/s12885-023-10709-5

**Published:** 2023-03-15

**Authors:** Yun Du, Xia Yu, Ellen T. Chang, Shifeng Lian, Biaohua Wu, Fugui Li, Bing Chu, Kuangrong Wei, Jiyun Zhan, Xuejun Liang, Weimin Ye, Mingfang Ji

**Affiliations:** 1 Cancer Research Institute of Zhongshan City, Zhongshan City People’s Hospital, Zhongshan, 528400 People’s Republic of China; 2 Department of Medical Epidemiology and Biostatistics, Karolinska Institutet, Stockholm, 17177 Sweden; 3 Department of Epidemiology and Biostatistics, University of California, California, USA; 4 Unit of Integrative Epidemiology, Institute of Environmental Medicine, Karolinska Institutet, Stockholm, 17177 Sweden; 5 Department of Pathology, Zhongshan City People’s Hospital, Zhongshan, 528400 People’s Republic of China; 6 Xiaolan Public Health Service Center, Zhongshan, 528400 People’s Republic of China

**Keywords:** Primary liver cancer, Epstein-Barr virus, Antibody, Nested case-control study, Population-based, Epidemiology

## Abstract

**Background:**

We aimed to investigate associations between pre-diagnostic anti-Epstein-Barr virus (EBV) antibodies, including interactions with hepatitis B virus (HBV), and risk of primary liver cancer in southern China.

**Methods:**

In a population-based nested case-control study, we measured pre-diagnostic immunoglobulin A (IgA) against EBV nuclear antigen 1 (EBNA1) and viral capsid antigen (VCA) in 125 primary liver cancer cases and 2077 matched controls. We also explored the interaction between HBV surface antigen (HBsAg) and anti-EBV antibodies.

**Results:**

Participants with positive EBNA1-IgA, positive VCA-IgA or single-positive anti-EBV antibodies had two-fold odds of developing liver cancer, compared with seronegative subjects. The odds ratios (ORs) between the relative optical density of EBNA1-IgA and VCA-IgA and primary cancer, controlling for age and HBsAg, were 1.59 (95% confidence interval (CI): 1.17, 2.14) and 1.60 (95% CI: 1.07, 2.41), respectively. Subjects with both HBsAg and anti-EBV antibody seropositivity were at 50-fold increased risk compared with those negative for both biomarkers (OR: 50.67, 95% CI: 18.28, 140.46), yielding a relative excess risk due to interaction of 30.81 (95% CI: 3.42, 114.93).

**Conclusion:**

Pre-diagnostic seropositivity for EBNA1-IgA and/or VCA-IgA was positively associated with primary liver cancer risk, especially in combination with HBsAg positivity. EBV may interact with HBV in the development of primary liver cancer, and anti-EBV antibodies might be potential biomarkers for primary liver cancer in this high-risk population.

**Supplementary Information:**

The online version contains supplementary material available at 10.1186/s12885-023-10709-5.

## Background

Epstein-Barr virus (EBV) is a double-stranded DNA virus and the first discovered human tumour virus, infecting over 90% of the total human population [1]. Primary infection typically occurs before 5 years of age and results in asymptomatic life-long infection, mainly in B cells and epithelial cells. However, a subset of the population develops EBV-related tumours, which include nasopharyngeal carcinoma (NPC), gastric cancer, Burkitt lymphoma, and Hodgkin lymphoma [2].

In 2020, with 906,000 incident cases and 830,000 deaths, primary liver cancer ranked as the sixth most common cancer and the third leading cause of death from cancer worldwide [3]. In China, approximately 410,038 incident primary liver cancer cases and 391,152 liver cancer deaths occurred in 2020 [4], making liver cancer the second most common malignancy among males and the sixth most common among females in some areas, such as Zhongshan City in southern China [5, 6].

The main risk factors for primary hepatocellular carcinoma (HCC), the main type of primary liver cancer, are chronic infection with hepatitis B virus (HBV) and hepatitis C virus (HCV), heavy alcohol drinking, diabetes, and aflatoxin B1 exposure. In China, nearly one-tenth of the population between ages 1 and 59 years are chronic carriers of HBV, which confers a 25–40% lifetime risk of HCC [7]. Accordingly, approximately 80% of HCCs in China occur among chronic HBV carriers [8, 9].

The role of EBV in HCC remains unclear and may vary among populations [10–13]. To date, no prospective studies have examined the associations between pre-diagnostic anti-EBV antibodies, with and without HBV infection, and the risk of primary liver cancer in southern China. Given that both chronic HBV infection and NPC, an EBV-related malignancy, are endemic in southern China, these viruses may interact uniquely in the development of primary liver cancer in this high-risk population. Alternatively, EBV-HBV interactions that generally occur in other populations, but have not yet been reported in the literature, may be more readily detected in southern China, given the enhanced statistical power from the relatively high prevalences of chronic HBV infection and immunoglobulin A (IgA) seropositivity for Epstein-Barr nuclear antigen 1 (EBNA1) and viral capsid antigen (VCA). Therefore, we investigated the role of pre-diagnostic EBV serology in primary liver cancer risk using a nested, prospective case-control study arising within a well-established, population-based NPC screening cohort in southern China.

## Methods

### Study population

The present study is a nested case-control study based on a population-based prospective cohort for NPC screening in Xiaolan town, Zhongshan City, southern China, between 2009 and 2017. Details of cohort recruitment were previously described [14]. Briefly, eligible participants were aged 30–59 years and residing in the study area. In total, 39,409 participants completed the initial screening. After excluding subjects with ages outside 30–59 years (*n* = 3585), those with missing birth date (*n* = 61), duplicate subjects (*n* = 232), and those with missing identity (*n* = 58) or sex (*n* = 9), 35,653 participants remained in the cohort. As of 30 June 2021, 127 confirmed incident primary liver cancer cases (presenting as 10th version of International Classification Disease C22) were identified through linkage to the cancer registry of Zhongshan City – two cases were excluded due to diagnosis prior to the baseline study examination, leaving 125 (83 image-based and 42 pathological-based) confirmed primary liver cancer cases. The index date for cases was the date of diagnosis.

**Fig. 1:**
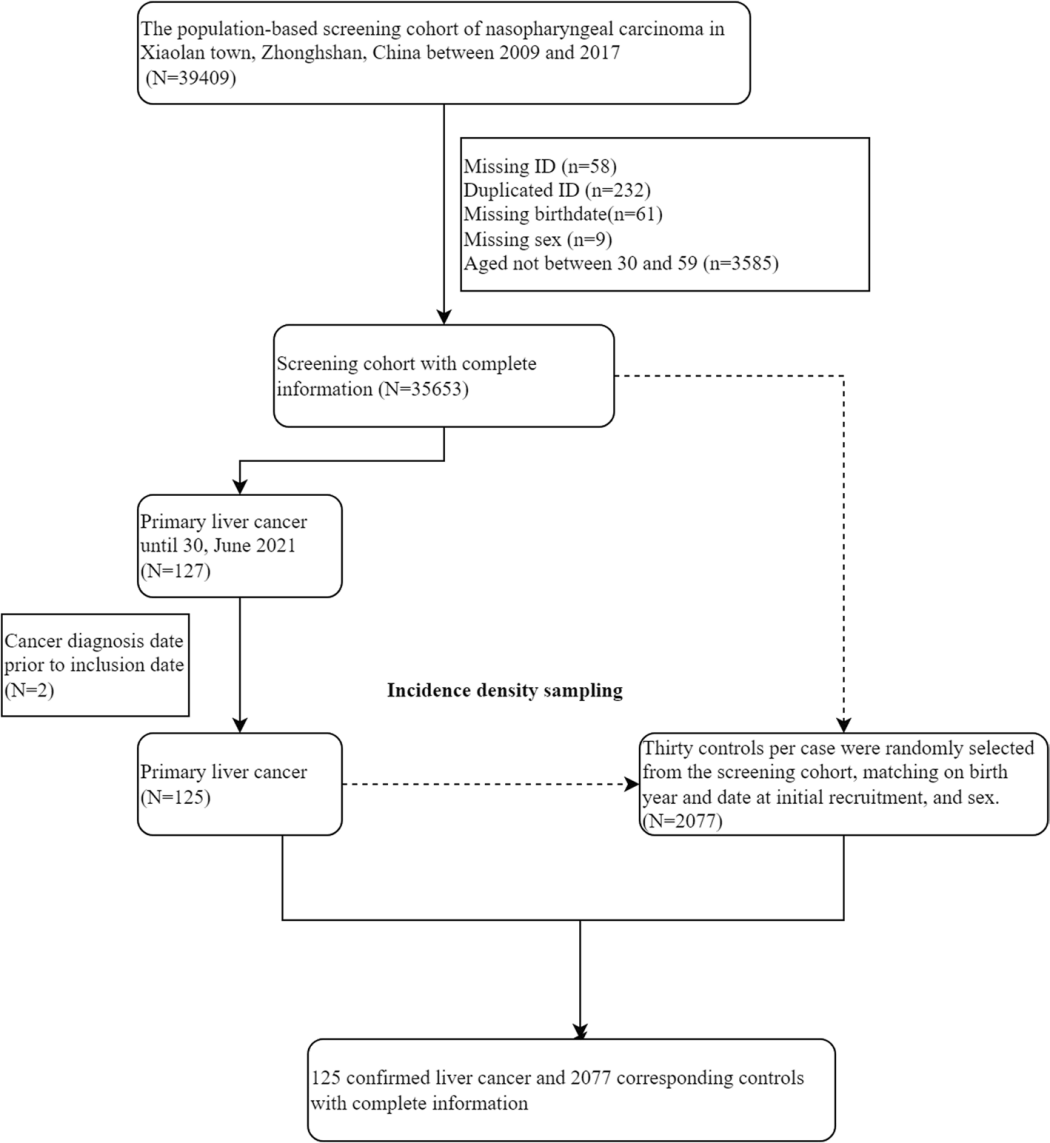
Flow chart of enrollment of study population. Abbreviation: ID, identity

For each case, we used risk-set sampling to randomly select 30 controls within the screening cohort who were alive on the case index date, did not migrate out of Zhongshan City, and were not diagnosed with primary liver cancer before the case index date on birth year, date of initial screening, and sex. Five cases were not matched to controls exactly on birth year; therefore, we matched three cases to controls within 1 year, and two cases within 8 years of the birth year **(**Fig. 1**)**.

### Exposure

Screening cohort participants donated 6 mL of whole peripheral blood at study enrollment and during cohort follow-up. The current analysis was restricted to the initial (earliest) blood sample. EBNA1-IgA (Zhongshan Bio-Tech Company, Zhongshan, China) and VCA-IgA (UROIMMUNAG, Lübeck, Germany) were measured in sera using relative optical density (rOD) by enzyme-linked immunosorbent assay (ELISA). According to the manufacturers’ instructions, antibodies in sera were classified as positive (rOD ≥1) or negative (rOD < 1). In addition, we divided the participants into three groups: double-negative (i.e., negative for both EBNA1-IgA and VCA-IgA), single-positive (i.e., positive for either EBNA1-IgA or VCA-IgA, but not both), and double-positive (i.e., positive for both EBNA1-IgA and VCA-IgA).

### Covariates

HBsAg in serum was detected by ELISA following the manufacturer’s instructions (Autobio Diagnostics Co., China). Samples were collected and tested in 2012 when a mass liver cancer screening program was launched in Zhongshan City [9]. Because chronic HBV infection is nearly universally acquired at birth or during early childhood in southern China [15], HBsAg seropositivity in adulthood generally reflects long-term chronic infection.

### Statistical analysis

The distributions of anti-EBV antibodies in primary liver cancer cases and controls were compared by Wilcoxon rank-sum tests. To evaluate the association between pre-diagnostic EBV seropositivity and primary liver cancer risk, we performed conditional logistic regression to estimate odds ratios (ORs) and 95% confidence intervals (CIs) with or without adjustment for age at enrollment (in years) as a continuous variable and HBsAg status (positive or negative) in matched case-control analyses accounting for risk sets.

Joint associations of HBV and EBV with primary liver cancer risk were tested by cross-classifying HBsAg status and anti-EBV antibody status as positive or negative (four categories: both positive, both negative, or either HBsAg or anti-EBV antibody-positive), and then testing for multiplicative interaction [16] or additive interaction by calculating relative excess risk due to interaction (RERI) [17, 18]. Only the 72 primary liver cancer cases and 953 matched controls with pre-diagnosis information on HBsAg status were included in this analysis.

### Sensitivity analysis

To rule out a reverse-causal effect of preclinical liver cancer on anti-EBV antibodies, we performed a sensitivity analysis restricted to cases with blood samples for anti-EBV antibodies obtained at least 2 years before diagnosis, and their corresponding matched controls.

All statistical tests were two-sided, and *P* values less than 0.05 were considered statistically significant. Data cleaning was performed with SAS (Version 9.4, SAS Institute, Inc., North Carolina, USA) and statistical analyses with R (Version 4.0.3).

## Results

### Study population characteristics

Among the 125 confirmed incident primary liver cancer cases (Table 1), 84.8% (106/125) were males; 58.4% (73/125) were aged between 50 and 59 at diagnosis; 88.8% (111/125) were HCC. Seventy-six cases had HBsAg tests, including four who were tested after cancer diagnosis (Fig. S1); 80.2% (61/76) of cases were positive for HBsAg. Characteristics of the 72 cases with pre-diagnosis HBsAg results and the 49 cases without HBsAg results did not show systematic differences (Table S1). For EBV serology, 9.6% (12/125) of cases and 4.9% (102/2077) of controls were EBNA1-IgA-positive, while 12.8% (16/125) of cases and 7.2% (150/2077) of controls were VCA-IgA-positive. Two cases (1.6%) and 20 controls (1.0%) were seropositive for both anti-EBV antibodies. The median time between anti-EBV antibody testing and cancer diagnosis was 4.74 years (range: 0.08, 110.2), and that for HBsAg testing was 3.45 years (range: − 0.74, 8.31) years (Fig. S1).

**Table 1:** Baseline characteristics of primary liver cancer cases and matched controls

Characteristics	Cases (***N*** = 125)	Controls (***N*** = 2077)	Total (***N*** = 2202)
Sex, N (%)			
Female	19 (15.2%)	445 (21.4%)	464 (21.1%)
Male	106 (84.8%)	1632 (78.6%)	1738 (78.9%)
Age at recruitment, N (%)			
30 ~ 39	14 (11.2%)	236 (11.4%)	250 (11.4%)
40 ~ 49	38 (30.4%)	655 (31.5%)	693 (31.5%)
50 ~ 59	73 (58.4%)	1186 (57.1%)	1259 (57.2%)
Classification, N (%)			
HCC	111 (88.8%)		
ICC	11 (8.8%)		
Others/Unknown	3 (2.4%)		
HBsAg, N (%)			
Negative,	15 (12.0%)	1135 (54.6%)	1150 (52.2%)
Positive	61 (48.8%)	250 (12.0%)	311 (14.1%)
Missing	49 (39.2%)	692 (33.3%)	741 (33.7%)
EBNA1-IgA, N (%)			
Negative	113 (90.4%)	1975 (95.1%)	2088 (94.8%)
Positive	12 (9.6%)	102 (4.9%)	114 (5.2%)
EBNA1-IgA, rOD			
Median (Min, Max)	0.21 (0.00, 8.18)	0.17 (− 0.02, 5.59)	0.17 (− 0.02, 8.18)
VCA-IgA, N (%)			
Negative	109 (87.2%)	1927 (92.8%)	2036 (92.5%)
Positive	16 (12.8%)	150 (7.2%)	166 (7.5%)
VCA-IgA, rOD			
Median (Min, Max)	0.39 (0.01, 2.51)	0.27 (−0.02, 4.01)	0.28 (− 0.02, 4.01)
Combination of VCA-IgA and EBNA1-IgA, N (%)			
Double-negative	99 (79.2%)	1845 (88.8%)	1944 (88.3%)
Double-positive	2 (1.6%)	20 (1.0%)	22 (1.0%)
Single-positive	24 (19.2%)	212 (10.2%)	236 (10.7%)

*Abbreviations*: *rOD* Relative optical density, *EBV* Epstein-Barr virus, *EBNA1* Epstein–Barr nuclear antigen 1, *VCA* Viral capsid antigen, *IgA* Immunoglobulin A, *HBsAg* Hepatitis B virus surface antigen

### Associations between anti-EBV antibodies and primary liver cancer

**Fig. 2:**
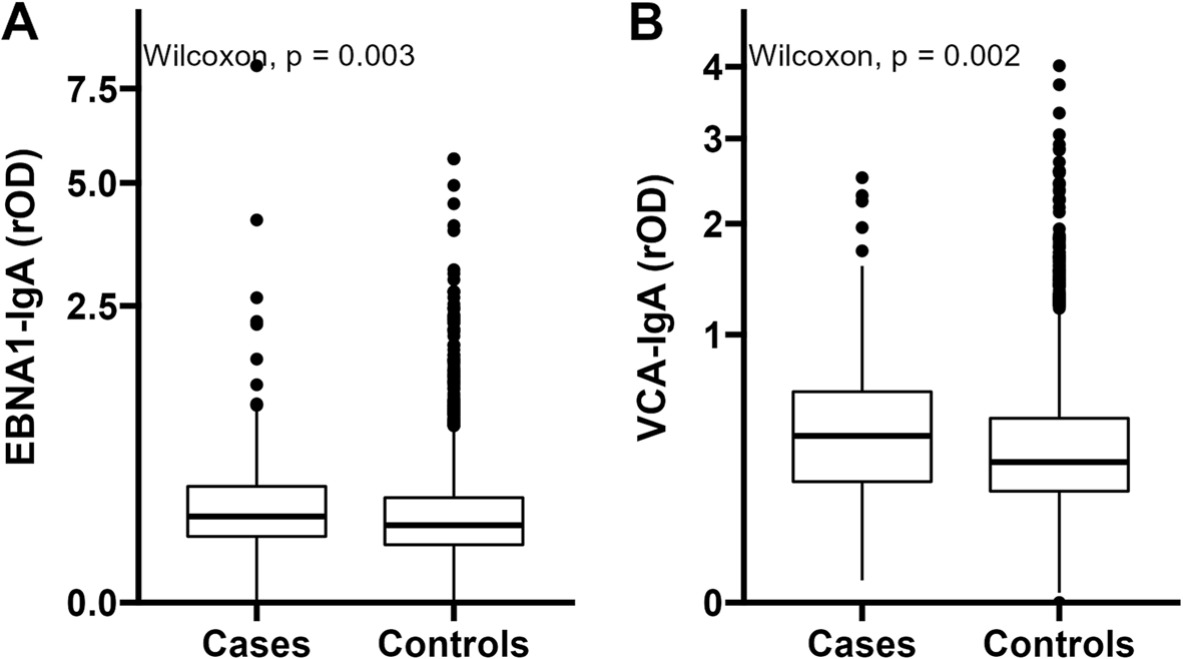
Boxplots of the distribution of rOD of EBNA1-IgA (**A**) and VCA-IgA (**B**) in primary liver cancer cases and controls. Differences were compared by Wilcoxon rank-sum test. Abbreviations: EBNA1: Epstein-Barr nuclear antigen 1; VCA: Viral capsid antigen; IgA: Immunoglobulin A; rOD: relative optical density

The distributions of EBNA1-IgA (*p* = 0.003) and VCA-IgA (*p* = 0.002) were significantly different between cases and controls (Fig. 2). Participants with positive EBNA1-IgA (adjusted OR: 2.19, 95% CI: 1.06, 4.51), positive VCA-IgA (adjusted OR: 1.93, 95% CI: 1.03, 3.65) or single-positive anti-EBV (adjusted OR: 2.00, 95% CI: 1.17, 3.42) antibodies had around two-fold odds of developing liver cancer, compared with seronegative subjects in adjusted models (Table 2). The age-adjusted ORs controlled for rOD of EBNA1-IgA and VCA-IgA were 1.52 (95% CI: 1.17, 1.98) and 1.63 (95% CI: 1.13, 2.34), respectively (Table 2).

**Table 2:** Associations of anti-EBV antibodies with odds of primary liver cancer risk

	Cases (***N*** = 125)	Controls (***N*** = 2077)	Crude OR (95% CI)	Adjusted OR (95% CI)^a^	Adjusted OR (95% CI)^b^
EBNA1-IgA					
Negative	113 (90.4%)	1975 (95.1%)	ref	ref	ref
Positive	12 (9.6%)	102 (4.9%)	**2.06 (1.08,3.93)**	**2.09 (1.09,4.00)**	**2.19 (1.06,4.51)**
EBNA1-IgA, rOD			**1.51 (1.16,1.96)**	**1.52 (1.17,1.98)**	**1.59 (1.17,2.14)**
VCA-IgA					
Negative	109 (87.2%)	1927 (92.8%)	ref	ref	ref
Positive	16 (12.8%)	150 (7.2%)	**1.96 (1.10,3.48)**	**1.99 (1.12,3.53)**	**1.93 (1.03,3.65)**
VCA-IgA, rOD			**1.61 (1.11,2.31)**	**1.63 (1.13,2.34)**	**1.60 (1.07,2.41)**
Combination of VCA-IgA and EBNA1-IgA					
Double-negative	99 (79.2%)	1845 (88.8%)	ref	ref	ref
Double-positive	2 (1.6%)	20 (1.0%)	1.97 (0.44,8.78)	1.93 (0.43,8.60)	3.03 (0.65,14.23)
Single-positive	24 (19.2%)	212 (10.2%)	**2.16 (1.33,3.52)**	**2.21 (1.35,3.61)**	**2.00 (1.17,3.42)**

*Abbreviations*: *EBV* Epstein-Barr virus, *rOD* Relative optical density, *EBNA1* Epstein–Barr nuclear antigen 1, *VCA* Viral capsid antigen, *IgA* Immunoglobulin A, *HBsAg* Hepatitis B virus surface antigen

^a^OR was adjusted by age (continuous) at initial recruitment

^b^OR was adjusted by age (continuous) at initial recruitment and HBsAg

Subgroup analysis (Table 3) showed that rOD of EBNA1-IgA remained associated with increased risk of primary liver cancer among males (adjusted OR: 1.55, 95% CI: 1.13, 2.14) and with increased risk of HCC specifically (adjusted OR: 1.51, 95% CI: 1.10, 2.09). Additional stratified associations between binary anti-EBV antibodies and primary liver cancer risk by age, sex and classification are presented in Tables S2 and S3. There was no significant difference between binary anti-EBV antibodies and primary liver cancer risk both in female and male strata.

**Table 3:** Associations between rOD of anti-EBV antibodies and primary liver cancer risk by sex, age and classification

			EBNA1-IgA			VCA-IgA		
Subgroups	Cases	Controls	Crude OR (95% CI)	Adjusted OR (95% CI)^a^	Adjusted OR (95% CI)^b^	Crude OR (95% CI)	Adjusted OR (95% CI)^a^	Fully adjusted OR (95% CI)^b^
Sex								
Female	19 (4.1%)	445 (95.9%)	2.02 (0.96,4.26)	2.10 (0.99,4.48)	2.14 (0.95,4.83)	1.70 (0.86,3.35)	1.71 (0.87,3.36)	1.57 (0.78,3.18)
Male	106 (6.1%)	1632 (93.9%)	**1.46 (1.11,1.92)**	**1.47 (1.11,1.94)**	**1.55 (1.13,2.14)**	**1.57 (1.02,2.42)**	**1.60 (1.04,2.45)**	1.61 (0.98,2.65)
Age at recruitment, years								
30 ~ 39	14 (5.6%)	236 (94.4%)	**5.95 (1.54,22.92)**	**5.68 (1.41,22.92)**	**5.34 (1.29,22.14)**	1.52 (0.41,5.69)	1.54 (0.39,6.13)	1.59 (0.41,6.19)
40 ~ 49	38 (5.5%)	655 (94.5%)	**1.46 (1.01,2.11)**	**1.46 (1.01,2.10)**	**1.56 (1.04,2.32)**	**2.53 (1.38,4.65)**	**2.54 (1.38,4.65)**	**2.62 (1.34,5.12)**
50 ~ 59	73 (5.8%)	1186 (94.2%)	1.43 (0.96,2.11)	1.43 (0.95,2.14)	1.46 (0.88,2.41)	1.28 (0.77,2.13)	1.30 (0.78,2.15)	1.17 (0.64,2.12)
Classification								
HCC	111 (5.9%)	1777 (94.1%)	**1.45 (1.10,1.92)**	**1.46 (1.10,1.93)**	**1.51 (1.10,2.09)**	**1.66 (1.12,2.44)**	**1.68 (1.14,2.48)**	**1.66 (1.06,2.60)**
ICC	14 (4.7%)	281 (95.3%)	1.64 (0.83,3.25)	1.75 (0.85,3.59)	1.75 (0.82,3.76)	1.01 (0.28,3.70)	1.01 (0.28,3.65)	1.05 (0.30,3.68)

*Abbreviations*: *rOD* Relative optical density, *EBV* Epstein-Barr virus, *EBNA1* Epstein–Barr nuclear antigen 1, *VCA* Viral capsid antigen, *IgA* Immunoglobulin A, *HCC* Hepatocellular carcinoma, *ICC* intrahepatic cholangiocarcinoma, *HBsAg* Hepatitis B virus surface antigen

^a^OR was adjusted by age (continuous) at initial recruitment

^b^OR was adjusted by age (continuous) at initial recruitment and HBsAg

### Joint associations between HBsAg and anti-EBV antibodies

When HBsAg and anti-EBV antibody status were cross-classified, the strongest association with primary liver cancer risk was observed for both serologically positive HBsAg and anti-EBV antibodies. Specifically, the adjusted OR for subjects who were both HBsAg-positive and anti-EBV antibody-positive was 50.67 (95% CI: 18.28, 140.46). The RERIs of VCA-IgA (35.07, 95% CI: 2.99, 144.76) and anti-EBV antibodies (30.81, 95% CI: 3.42, 114.93) with HBsAg were both significant, whereas no multiplicative-scale interactions were detected (Table 4).

**Table 4:** Joint associations between pre-diagnostic anti-EBV antibodies and HBsAg on primary liver cancer risk

	Cases (***N*** = 72)	Controls (***N*** = 953)	Adjusted OR (95% CI)^a^	RERI	Multiplicative scale
HBsAg, EBNA1-IgA, VCA-IgA				**30.81 (3.42,114.93)**	1.50 (0.28,8.02)
HBsAg (−) & Anti-EBV Ab (−)	11 (15.3%)	678 (71.1%)	ref		
HBsAg (−) & Anti-EBV Ab (+)	3 (4.2%)	98 (10.3%)	1.77 (0.42,7.49)		
HBsAg (+) & Anti-EBV Ab (−)	44 (61.1%)	160 (16.8%)	**19.09 (8.87,41.07)**		
HBsAg (+) & Anti-EBV Ab (+)	14 (19.4%)	17 (1.8%)	**50.67 (18.28,140.46)**		
HBsAg, EBNA1-IgA				11.88 (−21.94,131.33)	0.51 (0.06,4.09)
HBsAg (−) & EBNA1-IgA (−)	12 (16.7%)	728 (76.4%)	ref		
HBsAg (−) & EBNA1-IgA (+)	2 (2.8%)	48 (5.0%)	3.13 (0.65,14.96)		
HBsAg (+) & EBNA1-IgA (−)	54 (75.0%)	168 (17.6%)	**23.10 (10.97,48.65)**		
HBsAg (+) & EBNA1-IgA (+)	4 (5.6%)	9 (0.9%)	**37.11 (8.55,160.99)**		
HBsAg, VCA-IgA				**35.07 (2.99,144.76)**	6.02 (0.45,81.45)
HBsAg (−) & VCA-IgA (−)	13 (18.1%)	714 (74.9%)	ref		
HBsAg (−) & VCA-IgA (+)	1 (1.4%)	62 (6.5%)	0.53 (0.05,5.43)		
HBsAg (+) & VCA-IgA (−)	48 (66.7%)	167 (17.5%)	**17.72 (8.72,36.03)**		
HBsAg (+) & VCA-IgA (+)	10 (13.9%)	10 (1.0%)	**49.97 (16.21,154.01)**		

*Abbreviations*: *EBV* Epstein-Barr virus, *EBNA1* Epstein-Barr nuclear antigen 1, *VCA* Viral capsid antigen, *IgA* Immunoglobulin A, *HBsAg* Hepatitis B surface antigen, *RERI* Relative excess risk due to interaction

^a^OR was adjusted for age (continuous) at initial recruitment

### Sensitivity analysis

Restricting to cases with anti-EBV antibodies measured at least 2 years before diagnosis (Tables S4, S5), the adjusted OR for positive EBNA1-IgA was 2.37 (95% CI: 1.03, 5.45) and that for positive VCA-IgA was 1.91 (95% CI: 0.93, 3.95) (Table S5).

## Discussion

We set out to investigate the association between pre-diagnostic anti-EBV antibodies, including their interactions with HBV, and the risk of primary liver cancer in southern China. This investigation showed that participants who were positive for EBNA1-IgA and/or VCA-IgA prior to diagnosis had a significant about one-time greater increase in subsequent liver cancer risk. The relative risk was substantially more pronounced when both pre-diagnostic anti-EBV antibodies and HBsAg were positive, and the additive interaction between anti-EBV antibodies and HBsAg was statistically significant, indicating a possible super-additive interaction between the two viruses.

VCA-IgA indicates previous infection or reactivation of EBV, while EBNA1-IgA may be relevant to the release of EBNA1-DNA complex from host cells [19]. Thus, our findings indicate that EBV reactivity may be associated with the promotion of primary liver cancer, perhaps in cooperation with HBV, particularly in a population where HBV is the leading cause of liver cancer [20].

Our findings are consistent with those from two cross-sectional studies in Japan [10, 11] where EBV BamHIW DNA fragments were detected by Southern blot in 37% (13/52) and 33% (56/118), respectively, of tumour tissues from HCC patients, although in-situ hybridization (ISH) for Epstein-Barr encoding region (EBER) was negative. The second study found a higher frequency of EBV DNA in HCV-antibody-positive HCC than HBsAg-positive HCC, suggesting a possible interaction between EBV and HCV in tumour development [10, 11]. One hospital-based study of 78 HCC cases in southern China did find EBV infection involved in HCC development but no combination effect with HBV infection [21].

Studies in other populations outside of East Asia, however, found conflicting results. These include a cross-sectional study of 41 HCC cases (16 of Asian ethnicity) in Log Angels, California, where only one case was positive for EBNA1-ISH, one positive for BamHI Z Epstein-Barr virus replication activator (Zta), and two positive for EBNA1-ISH in tumor tissues [22]. Three other cross-sectional studies of 31 HCC patients in New York [23], 82 HCC patients in Germany and the United Kingdom [12], and 16 HCC patients in the Netherlands [13] also did not detect EBV DNA or transcripts in liver tumor tissue specimens.

To resolve these apparently contradictory results, we cannot exclude that the EBV DNA or proteins reportedly detected in HCC tumors were expressed in infiltrating lymphocytes, but not liver cells. Alternatively, EBV may contribute to primary liver cancer development in East Asia and other regions where the majority of disease is caused by HBV or HCV, but play a lesser role in Europe and North America, where a smaller proportion of primary liver cancer is attributed to the hepatitis viruses [20].

### Strengths and limitations

To our knowledge, this study is the first to prospectively investigate whether pre-diagnostic anti-EBV antibodies are associated with primary liver cancer risk. The prospective design minimizes the potential for reverse causation. Moreover, the population-based setting broadens the generalizability of our results to the southern Chinese population and enables the interpretation of ORs as rate ratios due to the incidence density sampling approach [24, 25].

We also acknowledge the limitations of this study. First, we only have two-thirds of subjects has HBsAg, including 58% of cases (72 of 125) with pre-diagnosis HBsAg status. Comparing cases with pre-diagnostic HBsAg and without HBsAg information, we found that the characteristic did not vary significantly (Table S1). Second, due to a lack of information on tumor EBV status, we could not determine whether anti-EBV antibodies were associated with EBV-positive liver cancer cells, EBV-positive lymphocytes infiltrating tumor tissues, or the general humoral immune response to EBV infection. Third, we acknowledge that there might exist uncontrolled confounding due to lack of information of some risk factors, for instance, alcohol consumption and red meat intake. Finally, in this population of predominantly HBV-related primary liver cancer, we could not determine whether risk associations with anti-EBV antibodies varied across different liver cancer etiologies, such as HBV-positive, HCV-positive, alcohol-related, and nonalcoholic-fatty-liver-disease-related. We were also unable to address whether our findings are specific to the southern Chinese population, where chronic HBV infection and EBV-associated NPC are endemic, or whether they might be generalizable beyond this region.

## Conclusion

To summarise, we found positive associations between pre-diagnostic serological EBNA1-IgA and VCA-IgA and risk of primary liver cancer in southern China. The associations with anti-EBV antibodies increased when pre-diagnostic HBsAg was also positive. Our findings point to the need to further elucidate the potential etiological role of EBV in primary liver cancer by investigating whether anti-EBV antibodies are associated with EBV-positive liver cancer cells, EBV-positive infiltrating lymphocytes, or the general humoral immune response to EBV infection.

## Supplementary Information

**Additional file 1: Table S1.** Baseline characteristics of primary liver cancer cases with pre-diagnostic HBsAg and cases without HBsAg.** Table S2.** Associations of anti-EBV antibodies with odds of primary liver cancer risk stratified by sex and age at recruitment.** Table S3.** Associations of anti-EBV antibodies of primary liver cancer risk stratified by histological classification.** Table S4.** Baseline characteristics of primary liver cancer cases and matched controls, restricting to cases with anti-EBV samples collected at least two years before cancer diagnosis. **Table S5.** Associations of anti-EBV antibodies with odds of primary liver cancer risk, restricting to cases with anti-EBV samples collected at least two years before cancer diagnosis.** Fig. S1.** Years of tests for HBsAg and anti-EBV antibodies before primary cancer diagnosis.

## Data Availability

The datasets (excluding individual information) used and/or analysed during the current study are available from the corresponding author on reasonable request.

## Abbreviations

HCC: Hepatocellular carcinoma
ICC: Intrahepatic cholangiocarcinoma
EBV: Epstein-Barr virus
EBNA1: Epstein-Barr virus nuclear antigen 1
VCA: Viral capsid antigen
IgA: Immunoglobulin A
rOD: Relative optical density
HBV: Hepatitis B virus
HBsAg: Hepatitis B virus surface antigen
HCV: Hepatitis C virus
