# Supplementary material for: Pre-diagnostic anti-EBV antibodies and primary liver cancer risk: a population-based nested case-control study in southern China
Source: BMC Cancer. 2023 Mar 15;23:250. doi: 10.1186/s12885-023-10709-5 (PMC10015780; doi:10.1186/s12885-023-10709-5)
Supplement: Supplementary file 1 — Additional file 1: Table S1. Baseline characteristics of primary liver cancer cases with pre-diagnostic HBsAg and cases without HBsAg. Table S2. Associations of anti-EBV antibodies with odds of primary liver cancer risk stratified by sex and age at recruitment. Table S3. Associations of anti-EBV antibodies of primary liver cancer risk stratified by histological classification. Table S4. Baseline characteristics of primary liver cancer cases and matched controls, restricting to cases with anti-EBV samples collected at least two years before cancer diagnosis. Table S5. Associations of anti-EBV antibodies with odds of primary liver cancer risk, restricting to cases with anti-EBV samples collected at least two years before cancer diagnosis. Fig. S1. Years of tests for HBsAg and anti-EBV antibodies before primary cancer diagnosis. [file 12885_2023_10709_MOESM1_ESM.docx]

**Title:** Pre-diagnostic anti-EBV antibodies and primary liver cancer risk: a population-based nested case-control study in southern China

Yun Du^1, 2, ǂ^ (0000-0001-8689-5375), Xia Yu^2, ǂ^, Ellen T. Chang^3, ǂ^, Shifeng Lian^4^, Biaohua Wu^2^, Fugui Li^2^, Bing Chu^5^, Kuangrong Wei^2^, Jiyun Zhan^6^, Xuejun Liang^6^, Weimin Ye^1, §^ (0000-0002-6859-4648), Mingfang Ji^2, §^ (0000-0003-4727-2858)

1, Department of Medical Epidemiology and Biostatistics, Karolinska Institutet, Stockholm 17177, Sweden;

2, Cancer Research Institute of Zhongshan City, Zhongshan City People’s Hospital, Zhongshan 528400, People’s Republic of China;

3, Department of Epidemiology and Biostatistics, University of California, San Francisco, United States;

4, Unit of Integrative Epidemiology, Institute of Environmental Medicine, Karolinska Institutet, Stockholm 17177, Sweden;

5, Department of Pathology, Zhongshan City People’s Hospital, Zhongshan 528400, People’s Republic of China;

6, Xiaolan Public Health Service Center, Zhongshan 528400, People’s Republic of China;

Correspondence to: Weimin Ye and Mingfang Ji.

^ǂ^ Yun Du, Xia Yu and Ellen T. Chang contributed equally.

^§^ Mingfang Ji and Weimin Ye contributed equally.

# Table S1

Table S1. Baseline characteristics of primary liver cancer cases with pre-diagnostic HBsAg and cases without HBsAg

| **Characteristics** | **Cases with pre-diagnostic HBsAg   (N= 72)** | **Cases without HBsAg   (N= 49)** | **Total   (N=121)** | **P^†^** |
| --- | --- | --- | --- | --- |
| Sex, N (%) |  |  |  | 0.69 |
| Female | 9 (12.5%) | 9 (18.4%) | 18 (14.9%) |  |
| Male | 63 (87.5%) | 40 (81.6%) | 103 (85.1%) |  |
| Age at recruitment, N (%) |  |  |  | 0.16 |
| 30~39 | 4 (5.6%) | 10 (20.4%) | 14 (11.6%) |  |
| 40~49 | 22 (30.6%) | 15 (30.6%) | 37 (30.6%) |  |
| 50~59 | 46 (63.9%) | 24 (49.0%) | 70 (57.9%) |  |
| Classification, N (%) |  |  |  | 0.92 |
| HCC | 65 (90.3%) | 42 (85.7%) | 107 (88.4%) |  |
| ICC | 6 (8.3%) | 5 (10.2%) | 11 (9.1%) |  |
| Others/Unknown | 1 (1.4%) | 2 (4.1%) | 3 (2.5%) |  |
| EBNA1-IgA, N (%) |  |  |  | 0.78 |
| Negative | 66 (91.7%) | 43 (87.8%) | 109 (90.1%) |  |
| Positive | 6 (8.3%) | 6 (12.2%) | 12 (9.9%) |  |
| EBNA-IgA, rOD |  |  |  | 0.80 |
| Median (Min, Max) | 0.21 (0.03, 2.20) | 0.21 (0.00, 8.18) | 0.21 (0.00, 8.18) |  |
| VCA-IgA, N (%) |  |  |  | 0.72 |
| Negative | 61 (84.7%) | 44 (89.8%) | 105 (86.8%) |  |
| Positive | 11 (15.3%) | 5 (10.2%) | 16 (13.2%) |  |
| VCA-IgA, rOD |  |  |  | 0.28 |
| Median (Min, Max) | 0.40 (0.01, 2.51) | 0.35 (0.07, 2.31) | 0.37 (0.01, 2.51) |  |
| Combination of VCA-IgA and EBNA-IgA, N (%) |  |  |  | 0.37 |
| Double-negative | 55 (76.4%) | 40 (81.6%) | 95 (78.5%) |  |
| Double-positive | 17 (23.6%) | 7 (14.3%) | 24 (19.8%) |  |
| Single-positive | 0 (0%) | 2 (4.1%) | 2 (1.7%) |  |
| Abbreviations: rOD, relative optical density; EBV, Epstein-Barr virus; EBNA1: Epstein–Barr nuclear antigen 1; VCA: viral capsid antigen; IgA: Immunoglobulin A; HBsAg: hepatitis B virus surface antigen. | | | | |
| ^†^P values across continuous variables were derived by Kruskal-Wallis test while categorical variables by Fisher's exact test. | | | | |

# Table S2

Table S2. Associations of anti-EBV antibodies with odds of primary liver cancer risk stratified by sex and age at recruitment

| **Subgroups** | **Anti-EBV antibodies** | **Cases** | **Controls** | **Crude OR (95% CI)** | **Adjusted OR (95% CI)^†^** | **Adjusted ORs (95% CI)^††^** |
| --- | --- | --- | --- | --- | --- | --- |
| Sex |  |  |  |  |  |  |
| Female | EBNA1-IgA |  |  |  |  |  |
|  | Negative | 17 (89.5%) | 428 (96.2%) | ref | ref | ref |
|  | Positive | 2 (10.5%) | 17 (3.8%) | 3.38 (0.70,16.21) | 3.45 (0.72,16.52) | 2.70 (0.52,14.08) |
| Male | EBNA1-IgA |  |  |  |  |  |
|  | Negative | 96 (90.6%) | 1547 (94.8%) | ref | ref | ref |
|  | Positive | 10 (9.4%) | 85 (5.2%) | 1.89 (0.93,3.84) | 1.92 (0.94,3.90) | 2.20 (0.98,4.94) |
| Sex |  |  |  |  |  |  |
| Female | VCA-IgA |  |  |  |  |  |
|  | Negative | 15 (78.9%) | 410 (92.1%) | ref | ref | ref |
|  | Positive | 4 (21.1%) | 35 (7.9%) | 3.02 (0.98,9.32) | 2.93 (0.95,9.08) | 2.39 (0.73,7.86) |
| Male | VCA-IgA |  |  |  |  |  |
|  | Negative | 94 (88.7%) | 1517 (93.0%) | ref | ref | ref |
|  | Positive | 12 (11.3%) | 115 (7.0%) | 1.72 (0.89,3.35) | 1.76 (0.90,3.42) | 1.84 (0.87,3.89) |
| Age at recruitment, years |  |  |  |  |  |  |
| 30~39 | EBNA1-IgA |  |  |  |  |  |
|  | Negative | 13 (92.9%) | 232 (98.3%) | ref | ref | ref |
|  | Positive | 1 (7.1%) | 4 (1.7%) | 4.13 (0.43,39.27) | 4.10 (0.42,40.42) | 6.21 (0.41,93.27) |
| 40~49 | EBNA1-IgA |  |  |  |  |  |
|  | Negative | 34 (89.5%) | 623 (95.1%) | ref | ref | ref |
|  | Positive | 4 (10.5%) | 32 (4.9%) | 2.41 (0.81,7.20) | 2.40 (0.80,7.18) | 2.53 (0.73,8.83) |
| 50~59 | EBNA1-IgA |  |  |  |  |  |
|  | Negative | 66 (90.4%) | 1120 (94.4%) | ref | ref | ref |
|  | Positive | 7 (9.6%) | 66 (5.6%) | 1.73 (0.73,4.06) | 1.73 (0.73,4.08) | 1.95 (0.75,5.09) |
| Age at recruitment, years |  |  |  |  |  |  |
| 30~39 | VCA-IgA |  |  |  |  |  |
|  | Negative | 12 (85.7%) | 227 (96.2%) | ref | ref | ref |
|  | Positive | 2 (14.3%) | 9 (3.8%) | 4.78 (0.87,26.19) | 5.27 (0.93,29.88) | 5.16 (0.89,30.04) |
| 40~49 | VCA-IgA |  |  |  |  |  |
|  | Negative | 32 (84.2%) | 615 (93.9%) | ref | ref | ref |
|  | Positive | 6 (15.8%) | 40 (6.1%) | **3.70 (1.42,9.63)** | **3.69 (1.41,9.65)** | **4.62 (1.58,13.46)** |
| 50~59 | VCA-IgA |  |  |  |  |  |
|  | Negative | 65 (89.0%) | 1085 (91.5%) | ref | ref | ref |
|  | Positive | 8 (11.0%) | 101 (8.5%) | 1.29 (0.58,2.86) | 1.25 (0.56,2.79) | 1.00 (0.40,2.48) |

# Table S3

Table S3. Associations of anti-EBV antibodies of primary liver cancer risk stratified by histological classification

| **Subgroups** | **Anti-EBV antibodies** | **Cases** | **Controls** | **Crude OR (95% CI)** | **Adjusted OR (95% CI)^†^** | **Adjusted OR (95% CI)^††^** |
| --- | --- | --- | --- | --- | --- | --- |
| HCC | EBNA1-IgA |  |  |  |  |  |
|  | Negative | 101 (91.0%) | 1689 (95.0%) | ref | ref | ref |
|  | Positive | 10 (9.0%) | 88 (5.0%) | 1.88 (0.93,3.82) | 1.90 (0.93,3.87) | 2.04 (0.91,4.56) |
| HCC | VCA-IgA |  |  |  |  |  |
|  | Negative | 97 (87.4%) | 1647 (92.7%) | ref | ref | ref |
|  | Positive | 14 (12.6%) | 130 (7.3%) | **1.89 (1.02,3.51)** | **1.92 (1.04,3.57)** | 1.90 (0.95,3.81) |
| ICC | EBNA1-IgA |  |  |  |  |  |
|  | Negative | 12 (85.7%) | 264 (94.0%) | ref | ref | ref |
|  | Positive | 2 (14.3%) | 17 (6.0%) | 2.42 (0.49,11.84) | 2.72 (0.53,13.90) | 2.45 (0.44,13.67) |
| ICC | VCA-IgA |  |  |  |  |  |
|  | Negative | 13 (92.9%) | 260 (92.5%) | ref | ref | ref |
|  | Positive | 1 (7.1%) | 21 (7.5%) | 1.02 (0.13,8.01) | 1.00 (0.13,7.90) | 0.98 (0.12,7.77) |
| Abbreviations: EBV, Epstein-Barr virus; EBNA1: Epstein–Barr nuclear antigen 1; VCA: viral capsid antigen; IgA: Immunoglobulin A; HCC, hepatocellular carcinoma; ICC: intrahepatic cholangiocarcinoma; HBsAg: hepatitis B virus surface antigen. | | | | | | |
| ^†^OR was adjusted by age (continuous) at initial recruitment. | | | | | | |
| ^††^OR was adjusted by age (continuous) at initial recruitment and HBsAg. | | | | | | |

# Table S4

Table S4. Baseline characteristics of primary liver cancer cases and matched controls, restricting to cases with anti-EBV samples collected at least two years before cancer diagnosis

| **Characteristics** | **Cases   (N=96)** | **Controls   (N=1642)** | **Total   (N=1738)** |
| --- | --- | --- | --- |
| Sex, N (%) |  |  |  |
| Female | 15 (15.6%) | 364 (22.2%) | 379 (21.8%) |
| Male | 81 (84.4%) | 1278 (77.8%) | 1359 (78.2%) |
| Age at recruitment, N (%) |  |  |  |
| 30~39 | 7 (7.3%) | 99 (6.0%) | 106 (6.1%) |
| 40~49 | 34 (35.4%) | 617 (37.6%) | 651 (37.5%) |
| 50~59 | 55 (57.3%) | 926 (56.4%) | 981 (56.4%) |
| Classification, N (%) |  |  |  |
| HCC | 82 (85.4%) |  |  |
| ICC | 11 (11.5%) |  |  |
| Others/Unknown | 3 (3.1%) |  |  |
| HBsAg, N (%) |  |  |  |
| Negative | 14 (14.6%) | 894 (54.4%) | 908 (52.2%) |
| Positive | 52 (54.2%) | 188 (11.4%) | 240 (13.8%) |
| Missing | 30 (31.3%) | 560 (34.1%) | 590 (33.9%) |
| EBNA1-IgA, N (%) |  |  |  |
| Negative | 87 (90.6%) | 1560 (95.0%) | 1647 (94.8%) |
| Positive | 9 (9.4%) | 82 (5.0%) | 91 (5.2%) |
| EBNA1-IgA, rOD |  |  |  |
| Median (Min, Max) | 0.24 (0.00, 8.18) | 0.17 (-0.02, 5.59) | 0.18 (-0.02, 8.18) |
| VCA-IgA, N (%) |  |  |  |
| Negative | 83 (86.5%) | 1525 (92.9%) | 1608 (92.5%) |
| Positive | 13 (13.5%) | 117 (7.1%) | 130 (7.5%) |
| VCA-IgA, rOD |  |  |  |
| Median (Min, Max) | 0.38 (0.01, 2.51) | 0.27 (-0.02, 4.01) | 0.28 (-0.02, 4.01) |
| Combination of VCA-IgA and EBNA1-IgA, N (%) |  |  |  |
| Double-negative | 75 (78.1%) | 1459 (88.9%) | 1534 (88.3%) |
| Double-positive | 1 (1.0%) | 16 (1.0%) | 17 (1.0%) |
| Single-positive | 20 (20.8%) | 167 (10.2%) | 187 (10.8%) |
| Abbreviations: rOD, relative optical density; EBV, Epstein-Barr virus; EBNA1: Epstein–Barr nuclear antigen 1; VCA: viral capsid antigen; IgA: Immunoglobulin A; HBsAg: hepatitis B virus surface antigen. | | | |

# Table S5

Table S5. Associations of anti-EBV antibodies with odds of primary liver cancer risk, restricting to cases with anti-EBV samples collected at least two years before cancer diagnosis

|  | **Cases (N=96)** | **Controls (N=1642)** | **Crude OR (95% CI)** | **Adjusted OR (95% CI)^†^** | **Adjusted OR (95% CI)^††^** |
| --- | --- | --- | --- | --- | --- |
| EBNA1-IgA |  |  |  |  |  |
| Negative | 87 (90.6%) | 1560 (95.0%) | ref | ref | ref |
| Positive | 9 (9.4%) | 82 (5.0%) | **2.12 (1.02,4.38)** | 2.06 (0.98,4.34) | **2.37 (1.03,5.47)** |
| EBNA1-IgA, rOD |  |  | **1.55 (1.17,2.06)** | **1.51 (1.13,2.01)** | **1.67 (1.22,2.30)** |
| VCA-IgA |  |  |  |  |  |
| Negative | 83 (86.5%) | 1525 (92.9%) | ref | ref | ref |
| Positive | 13 (13.5%) | 117 (7.1%) | **2.11 (1.11,4.00)** | **2.16 (1.13,4.10)** | 1.91 (0.93,3.95) |
| VCA-IgA, rOD |  |  | **1.62 (1.09,2.42)** | **1.63 (1.09,2.43)** | 1.55 (0.98,2.45) |
| Combination of VCA-IgA and EBNA1-IgA |  |  |  |  |  |
| Double-negative | 75 (78.1%) | 1459 (88.9%) | ref | ref | ref |
| Double-positive | 1 (1.0%) | 16 (1.0%) | 1.29 (0.17,9.97) | 1.38 (0.18,10.71) | 1.75 (0.21,14.61) |
| Single-positive | 20 (20.8%) | 167 (10.2%) | **2.49 (1.45,4.28)** | **2.47 (1.43,4.27)** | **2.31 (1.25,4.26)** |
| Abbreviations: EBV, Epstein-Barr virus; rOD, relative optical density; EBNA1: Epstein–Barr nuclear antigen 1; VCA: viral capsid antigen; IgA: Immunoglobulin A; HBsAg: hepatitis B virus surface antigen. | | | | | |
| ^†^OR was adjusted by age (continuous) at initial recruitment. | | | | | |
| ^††^OR was adjusted by age (continuous) at initial recruitment and HBsAg. | | | | | |

# Figure S1


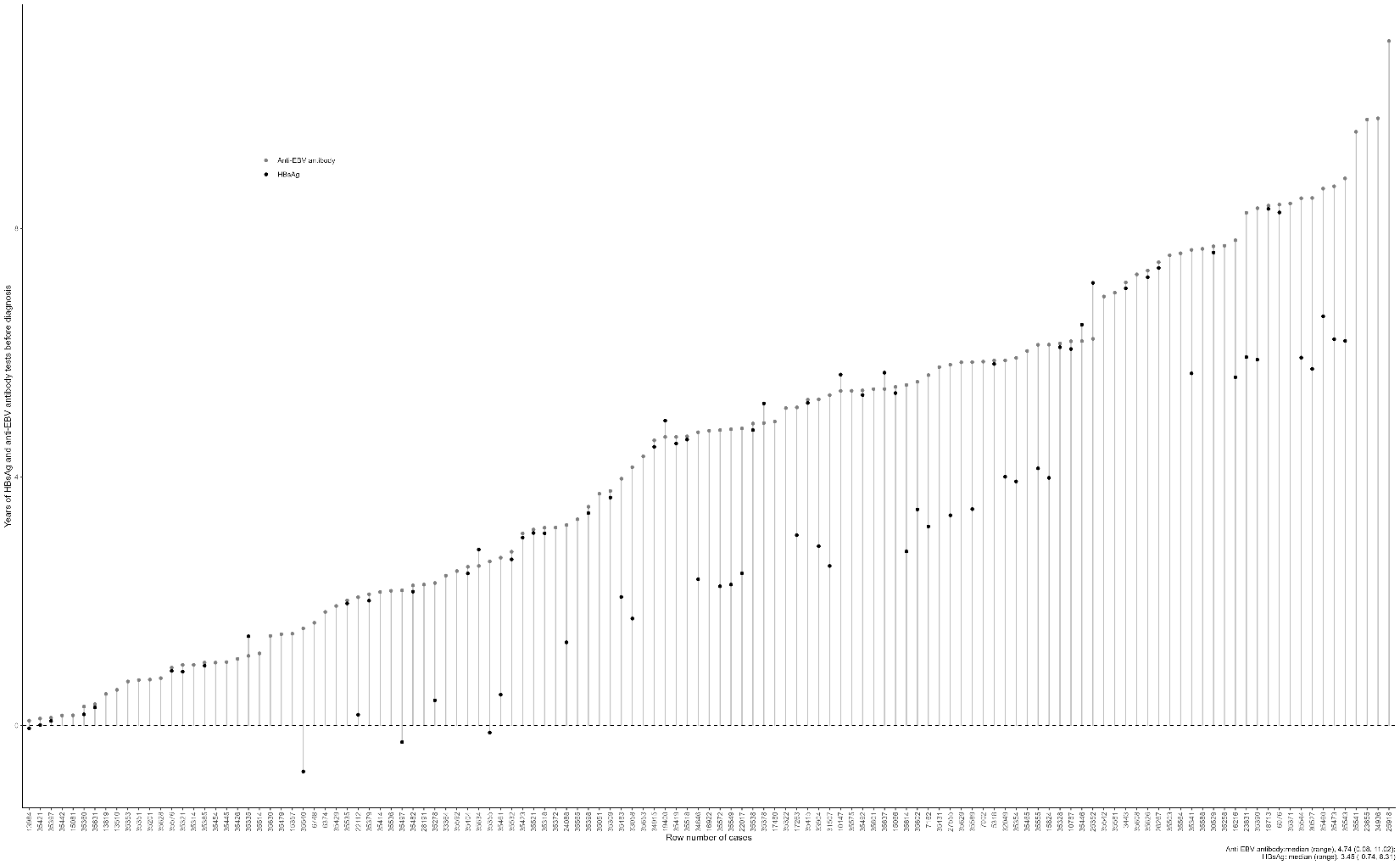


Figure S1: Years of tests for HBsAg and anti-EBV antibodies before primary cancer diagnosis

Abbreviations: EBV, Epstein-Barr virus; EBNA1: Epstein–Barr nuclear antigen 1; VCA: viral capsid antigen; IgA: Immunoglobulin A; HBsAg: hepatitis B virus surface antigen.
